# Stem Cells in Ovarian Cancer and Potential Therapies

**Published:** 2020-05-03

**Authors:** Elena Zuber, Diana Schweitzer, Dominick Allen, Seema Parte, Sham S. Kakar

**Affiliations:** 1 Department of Physiology, University of Louisville, Louisville, KY40202,; 2 Department of Biochemistry and Molecular Biology, University of Nebraska, Omaha, NE-68198-5870,; 3 James Graham Brown Cancer Center, University of Louisville, Louisville, KY 40202.

## INTRODUCTION

Ovarian cancer represents the fifth cause of deaths from cancer accounting to 21,750 new cases and 13,940 deaths expected in the USA in 2020 [1, 2]. Due to late diagnosis of ovarian cancer, the cancer cells are already disseminated to the peritoneal cavity at diagnosis and thus impose a serious clinical challenge [3–5]. Ovarian tumors may be divided into three types in particular: epithelial, germ cell, and stromal cells—epithelial carcinoma being the most abundant [6]. Furthermore, within epithelial tumors there are four subtypes, serous, endometrioid, clear cell, and mucinous—wherein serous tumors are the most lethal [6–8]. The first line treatment for ovarian cancer is cytoreductive surgery followed by chemotherapy mainly combination of carboplatin and paclitaxel [9, 10]. Initial response of this combination therapy is very high (70%), however after few treatments, patients develop cisplatin resistance leading to tumor relapse and recurrence [11, 12]. Recently, several investigators have indicated the presence of cancer stem cells (CSCs) also known as cancer initiating cells in tumors as putative entities responsible for cancer initiation and progression [13, 14]. These CSCs have been reported to be chemo- and radio- resistant, and ultimately leading to cancer recurrence [15–19]. Therefore, it is crucial to understand the biology of CSCs including their regulation in order to develop therapies that can target both the cancer cells and CSCs and thus provide highly effective therapy for the treatment of cancer. Present review article briefly covers the biology of different populations of CSCs in ovarian cancer based upon several reported CSC specific biomarkers and cell surface markers and potential therapies being developed recently to target CSCs.

## CANCER STEM CELLS

Cancer arises from a cell type within the tumors that can undergo self-renewal and promotes tumorigenesis—these cells are known as tumor initiating cells or cancer stem cells (CSCs). Various specific markers including but not limited to ALDH1/2, CD133, CD117, CD24, CD34, CD44, EpCAM, NANOG, OCT 3/4, LGR5 and LY6A have been reported and used in isolation and characterization of CSCs from ovarian cancer cell lines, ovarian cancer, and ascites collected from patients with recurrent ovarian cancer [20, 21]. Currently, it is accepted that CSCs are not only responsible for the development of chemotherapeutic and cytostatic resistances, but also for primary tumor growth, metastasis and tumor relapse [22–24].

In addition to their origin and morphologies, these malignant populations also vary in their biological behavior [24]. A tiny population of stem cells with embryonic characteristics from normal human ovaries [25–29] have been suggested as progenitors [28–30], however, this has yet to be elucidated. The high level of non-consistent gene mutations giving rise to heterogeneous populations making a daunting task in identifying a suitably effective target. Even though the existence of CSCs has been identified in a variety of tumors, their origin is not well understood. Owing to the common characteristics and self-renewal mechanisms shared between stem as well as CSCs, it is speculated that cancer may be originating from the transformation of normal tissue specific stem cells [31] i.e. ovarian stem cells in this instance. High levels of expression of several oncogenes and transforming genes in CSCs support the hypothesis that CSCs could be a result of transformation of normal stem cells present in adult tissues [31]. However, this hypothesis needs to be tested. Ovarian CSCs have been attributed with characteristics of self-renewal, tumor-initiation, growth, differentiation, drug resistance, and tumor relapse [31]. In this study an overlooked and unconventional role of PTTG1 as a marker of CSCs (in normal ovaries, benign, borderline, high grade tumors and ascites derived tumors) and its ability to modulate CSCs via the ovarian germline and stemness-related genes was dissected very intricately and reported for the first time by exploring the self-renewal and epithelial-mesenchymal transition pathways regulated by PTTG1.

Recently, our group have also identified and characterized ovarian stem cells and CSC compartments on basis of unique germline stem cell specific marker VASA with the help of co-expression studies. Non-proliferating and quiescent stem cell populations were identified in normal ovaries besides benign, borderline and high-grade ovarian tumors. Typically, two distinct stem like/cancer stem-like cells expressing various combination of markers were detected in the samples including normal ovaries [31–34]. In a quest towards identifying several heterogeneous CSC populations in ovarian tumors and metastatic ascites derived fluid, our group has extensively characterized these cells with the aid of several biomarkers and stemness associated genes. In one study, the germline stem cell marker associated with the normal ovarian stem cells was found to be co-expressed with most of the CSC specific surface markers with their prominent localization in the ovarian surface epithelium (OSE) layer and the adjacent ovarian cortex [33]. An interesting localization, distribution and predominance of specific combination of markers were detected across the normal ovaries, benign, borderline and high-grade ovarian tumor samples from patients. At the same time, other study revealed the proliferating and quiescent populations of CSCs with the aid of Ki67 within similar samples [31]. Further, we reported the exclusive localization of an oncogene PTTG in not only normal ovaries, benign, borderline and high-grade ovarian tumors but also the ascites fluid [31]. We reported for the first time a systematic expression profiling of various CSC markers and their co-expression with the oncogene PTTG. Our study highlighted that the stem cell specific self-renewal related and EMT specific pathways [31] executed CSC regulation by PTTG. An interesting network of stemness pathways governing the various CSC populations in ovarian tumors and similar cellular signaling networks operating in ascites derived CSCs was anticipated based on persistence of similar CSC populations. Hence, a thorough understanding of the stem cell contribution to tumor pathogenesis (ie. tumor initiation, dissemination and eventual culmination into therapy resistance) attributed to CSCs warrant a thorough investigation while not overlooking real time patient-level clinical scenario of treatment regimen administered. Some of the commonly identified cancer stem cells in ovarian cancer are listed in Table 1.

## POTENTIAL THERAPIES FOR OVARIAN CANCER

Ovarian cancers are difficult to treat due to their complexity and heterogeneity from patient to patient. Presence of CSCs in ovarian cancer and ascites are resistant to chemotherapy, and their amplification occurs upon treatment with currently used chemotherapies. Investigators have shown enrichment of these CSC populations when treated with chemotherapy, in vitro and in experimental models, resulting in increased drug resistance [34, 35].

ALDH1A1+ CSCs have been implicated in rendering resistance to the PARPi in BRCA2-mutated epithelial ovarian cancer [36]. A type I receptor tyrosine kinase-like orphan receptor (ROR1) expressed during embryogenesis and in several cancers is expressed in ovarian CSCs and contributes towards migration/invasion or spheroid formation in vitro and tumor engraftment in immune-deficient mice thus serving as a potential targetable molecule [37]. Recently propagation of primary ovarian CSCs specific for individual patient, followed by 3D hanging drop suspension culture to establish spheroidal cultures and xenografting in immuno-compromised mice for pre-clinical drug screening, especially for assessing patient-specific (personalized) responses to chemotherapeutics and for delving deeper into understanding therapeutic resistance from a CSC standpoint have been established. Such platforms may enable testing of CSC heterogeneity and differential suppression as well as enrichment of sensitive and resistant populations respectively [38]. Since phenomena of EMT, chemoresistance, tumor invasion, metastasis, recurrence and CSCs are inter-related, development of multi-targeted approaches in the future may help to eradicate various CSC populations expressing CD117, CD133, Notch and targeting of EMT/CSCs pathways such as PI3K/mTOR, JAK2/STAT3, DNA methyltransferase, p53 mediated apoptosis, EGFR/Stat3, TGF- β, Notch3/ERK etc. may prove highly promising. EMT targeting approaches to overcome resistance feature are worth pursuing and systematic evaluation of clinical efficacy in clinical trials are further warranted [39, 40]. In recent years, varied therapies are being tested to target CSCs in order to reduce or eliminate recurrence of cancer which are addressed in following sections.

### Targeting of Self-renewal signaling pathways

**Figure 1.**
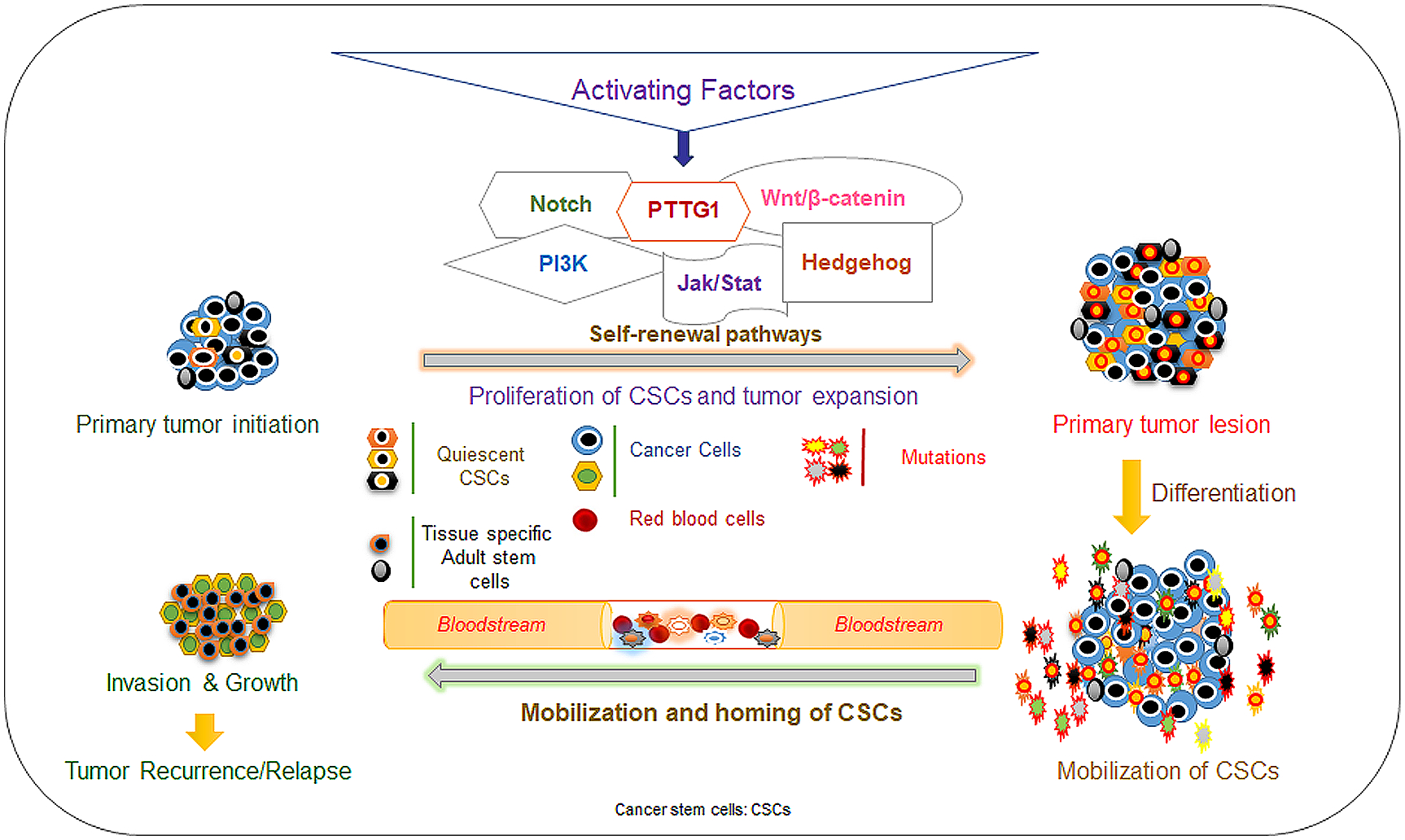
Schematic representation of various factors regulating the signaling pathways involved in cancer stem cell self-renewal, differentiation and metastasis.

Mutations, overexpression and dysregulation of several CSC self-renewal pathways have been implicated in the progression, metastasis, and recurrence of ovarian cancer. These pathways often lead to growth and maintenance of ovarian CSCs. Some of these pathways include Notch, PI3K/PTEN/AKT, JAK/STAT, Wnt/β-catenin, and Sonic Hedgehog (SHH) (Fig. 1). Current research has focused on these signaling pathways in the context of targeting of CSCs.

### Notch signaling pathway

The Notch signaling pathway is an evolutionarily conserved signaling cascade involved in embryonic development. Additionally, increased expression of Notch3 has correlated with poor prognosis in patients with ovarian cancer. It plays a critical role in maintenance, differentiation, proliferation, communication, and apoptosis of progenitor cells; and inextricably intertwined for many cancers, including ovarian cancer [41, 42]. More specifically, it contributes toward the maintenance of ovarian CSCs and their resistance to chemotherapy.

The protein coding gene Notch3 is amplified and overexpressed in the majority of high-grade serious ovarian carcinoma (HGSOC) [43]. The most highly expressed Notch3 ligand is Jagged1. This is expressed primarily in mesothelial cells in a tumor microenvironment, indicating that this pathway likely contributes to the signaling that regulates cell adhesion and tumor proliferation [42, 43]. Notch3 has also been shown to play a critical role in chemoresistance and stemness of ovarian CSCs, and it can be used as a prognostic indicator. Activation of Notch receptors leads to proteolytic cleavage of the intracellular Notch domain mediated by γ-secretase. This cleaved domain then translocates to the nucleus, where it regulates gene transcription [44]. It has been reported that overexpression of Notch3 results in increased expression of stem cell markers such as OCT4, SOX2, and NANOG. OCT4 promotes self-renewal of CSCs, while SOX2 is required for their maintenance [43]. Notch overexpression also causes enhanced expression of a transport protein (ABCG1) that increases chemo-resistance, specifically to platinum and carboplatin. In addition, it leads to expansion of the side population of CSCs, resulting in increased chemoresistance [43, 44].

Downstream effects of Notch were improved by knockdown of Notch3. Inhibition of Notch led to depletion of total number of ovarian CSCs along with increased tumor sensitivity to platinum [44]. Additionally, down-regulation of Jagged1 led to increased sensitivity to chemotherapy, further supporting the evidence that the Notch pathway contributes to stemness and chemoresistance [43]. One study used a γ-secretase inhibitor in conjunction with cisplatin to induce DNA damage, cell cycle arrest, and apoptotic cell death [42]. Notch3 protein when examined in primary and recurrent tumors from the same patients showed elevated expression levels in the recurrent tumors as compared to the primary tumors indicating its relationship with poor prognosis [43, 44], suggesting that the Notch pathway is a promising candidate for targeted therapies.

### Wnt/β-Catenin Pathway

Another important pathway relevant to both embryogenesis and ovarian cancer is the Wnt/β-catenin pathway. During embryogenesis, it regulates cell fate and is involved in the normal development of ovaries and fallopian tubes. In adults, it is critical for self-renewal in tissues, and plays a role in maintenance, quiescence, and chemoresistance of stem cells [45, 46]. Since, this pathway is so complex, its signaling appears to differ among different histotypes of ovarian cancer.

In its deactivated form, β-catenin is degraded within proteasomes. However, on activation of Wnt/β-catenin pathway, β-catenin is not phosphorylated and it is released from the destruction complex and translocates to the nucleus, where it acts as a transcription factor, thereby activating Wnt target genes – including those involved in stemness and chemoresistance [47–50]. These target genes include those that code for leucine-rich-repeat-containing G protein-coupled receptors (LGR), which is not only expressed in ovarian cancer but also in several cancer types [51]. LGR5 is a stem cell marker for ovarian stem cells and LGR6 is a cancer stem cell marker for fallopian tube, and expression of either one is a sign of elevated Wnt significantly [51–54]. Elevated Wnt signaling in tumors indicates that Wnt signaling is necessary for the expression of the stem cell factors that support tumor growth and maintenance [51]. Down-regulation of LGR5 has been found to be associated with lower CSC-like phenotypes and lower chemo-resistance properties in vitro. Additionally, silencing of LGR5 inhibited stemness and chemo-resistance in vivo, specifically through inhibition of the Wnt/β-catenin signaling pathway [53–55].

Evidence show that, when Wnt target genes are activated, a hypoxic niche leads to CD117 expression, leading to Akt activation and nuclear β-catenin expression, which induces a drug transporter that leads to chemoresistance [43]. Knockdown of CD117 by using specific siRNA results in lower numbers and size of ovarian CSC subpopulations and their pro-tumorigenic activity, indicating that CD117 contributes to the tumorigenic and chemoresistant properties of ovarian CSCs through Wnt activation [43]. This is further supported by the finding that CD117 overexpression leads to the upregulation of ATP-binding cassette G2 (ABCG2) in the Wnt/β-catenin pathway, enhancing chemoresistance. Similarly, expression of Wnt/β-catenin target genes and ligands were upregulated in chemoresistant ovarian cancer cells, and these cells showed high expression of CSC markers [54, 55]. More importantly, it was found that Wnt/β-catenin specific inhibitors sensitize ovarian CSCs to chemotherapy and reduce the number of CSC subpopulations, indicating that inhibition of this pathway can stifle CSC characteristics and enhance chemosensitivity [55].

Raghavan et al [56] found that tumor-associated macrophages are responsible for promoting metastasis, angiogenesis, and tumorigenesis within the tumor microenvironment, so these are likely to play a role in recurrence, metastasis, resistance, and preservation of stem-like phenotypes through Wnt signaling. These investigators also found that paracrine Wnt activation during interactions between CSCs and M2 macrophages make up a positive feedback loop, which likely contributes to phenotypes that are more aggressive. This evidence further supports that Wnt pathway as a potential target, particularly for the reduction/elimination of CSC and M2 macrophages in the tumor microenvironment. Another promising therapy was found to be theaflavin-3, 3’digallate (TF3). This compound was shown to inhibit ovarian CSCs by inducing apoptosis and cell cycle arrest and inhibiting angiogenesis through the Wnt/β-catenin pathway [57].

### Hedgehog Signaling Pathway

During embryogenesis, the Hedgehog pathway regulates tissue polarity and pattern along with stem cell development and maintenance [58]. This pathway consists of secreted ligands: Sonic hedgehog (Shh), Indian hedgehog (Ihh), and Desert hedgehog (Dhh); Hedgehog receptors: Patched (PTCH1, PTCH2) and Smoothened (SMO); and Gli transcription factors 1, 2, and 3 [58]. Activation of this pathway is associated with regulation of CSC phenotypes like self-renewal, differentiation, and tumor initiation [59]. This pathway can become disrupted in two ways in ovarian cancer: 1) disrupted by the overexpression of endogenous pathway ligands such as Shh; 2) disrupted through using inhibitor vismodegib [60].

It has been reported that ovarian CSCs exhibit increased intracellular Gli1 expression, which correlates with increased formation of spheroids with CSC properties. Gli1 has also been found to play a role in expression of ABC transporters ABCG1 and ABCG2 by directly binding to their promoter regions resulting in increased resistance to both cisplatin and paclitaxel in spheroid forming of ovarian cancer cells [61, 62]. Additionally, there is higher expression of SMO and Gli1 in borderline and malignant tumor tissues than in benign tumor and normal ovarian tissues [63–64], indicating their importance in progression of ovarian cancer.

Investigators found that the inhibition of the hedgehog pathway by cyclopamine resulted in a decrease in Gli concentrations followed by decreased cancer cell proliferation [65]. Furthermore, it was demonstrated that cyclopamine also inhibited tumor growth and impaired spheroid function. Exogenous expression of Gli was found to increase both cell proliferation and invasiveness by at least 200%, demonstrating the effect of Gli on ovarian cancer cell proliferation [66]. Hh pathway components SMO, PACH and GLI1 were reported to be activated in benign, borderline and malignant ovarian epithelial tumors and implicated its association with cisplatin resistance. [67–68]. This clearly implicates importance of Hedgehog signaling in malignancy and chemoresistance of ovarian CSCs. GDC-0449, a potent hedgehog inhibitor, is in the clinical phases for its application in treating ovarian cancer patients [68].

### PI3K/PTEN/AKT Pathway

Phosphatidylinositol 3-kinases (PI3K) is an enzyme involved generally in cell growth, proliferation, differentiation, motility, survival, and intracellular trafficking. It appears that the PI3K/PTEN/AKT is activated in approximately half of high-grade serious ovarian carcinoma (HGSOC), making it a potential therapeutic target for ovarian cancer. Additionally, high expression of activated Akt is associated with poor prognosis and survival, due to mutations that could occur in several components of this pathway [69, 70]

This pathway has been found to regulate enrichment of ovarian CSCs, maintenance of the stem cell (SC) phenotype, and chemoresistance. Ovarian spheroids show high expression of pAKT1 and low expression of PTEN, along with increased resistance to paclitaxel [71–74]. Additionally, inhibition of AKT1 results in decreased spheroid formation and migration. Furthermore, knockdown of AKT1 using siRNA led to loss of CSC marker expression, loss of spheroid formation, and loss of paclitaxel resistance [75, 76]. In cisplatin-resistant CSCs, AKT regulates expression of PPM1D, a gene that codes for a protein, which inhibits DNA damage and apoptotic response after DNA damage. Moreover, the down-regulation of AKT activity led to loss of PPM1D stability and an increase in its degradation [77, 78]. This, in turn, led to increased response to cisplatin, indicating the importance of this pathway in initiation and maintenance of CSCs and their chemoresistance, making this pathway an ideal target for ovarian cancer therapy.

PI3K/PTEN/AKT pathway also appears to play a crucial role through C-Kit, a well-known proto-oncogene of ovarian CSCs [79]. Its ligand, stem cell factor (SCF) can exist either in soluble form or as a transmembrane protein. SCF interacts with C-kit to regulate cell viability, proliferation, and differentiation. SCF was found in high levels in epithelial ovarian cancer, but it appears to be only membrane bound in tumor cells [80]. Both secretory and membrane bound forms of SCF are observed in tumor associated macrophages (TAM) and fibroblasts (TAF). Interestingly, in the circulating monocytes of healthy patients neither of these forms were observed. However, both forms were produced upon differentiation into macrophages, with M1 and M2 polarization having no effect [79]. Both of these isoforms were then able to activate the AKT pathway in cells with c-Kit receptor. This effect was also counteracted by imatinib, a tyrosine kinase inhibitor. These findings are thus an evidence of a juxtacrine/paracrine circuit in ovarian cancer that may exist and act through the AKT pathway [79].

### JAK/STAT Pathway

The JAK/STAT signaling pathway is a universally expressed intracellular signal transduction pathway and is involved in many crucial biological processes, including cell proliferation, differentiation, apoptosis, and immune regulation. JAK/STAT pathway also generally regulates differentiation, proliferation, and survival of stem cells. In ovarian cancer, the JAK/STAT pathway almost is always active, with no inhibition or regulation of its activity [80]. This pathway has been reported as an important pathway in maintaining CSC population especially OCT4 in ovarian cancer [80, 81]. There are four JAK family non-receptor tyrosine kinases, JAK1, JAK2, JAK3 and TYK2. It has been reported that JAK1, JAK2 and TYK2 are ubiquitously expressed, whereas JAK3 is predominantly expressed in hematopoietic cells. There are several types of STAT (STAT1, STAT2, STAT3, STAT4, STAT5a and b). During the activation of the signaling cascade, these JAK and STAT molecules assemble into homo- and hetero-dimers, or even into more complex multi-mers [81]. Jin [82] showed that activation of JAK/STAT3 pathway leads to increased tumorigenesis and metastasis ability, the transition of cancer stem cells (CSCs), and chemoresistance in cancer through the regulation of epithelial-mesenchymal transition (EMT) [82] inducing transcription factors such as Snail, Zeb1, JUNB, and Twist1 [84–86, 86]. Activation of JAK/STAT signaling is triggered by various hormones, cytokines (including the IL-6 family) [83], and growth factor through a variety of molecular mechanisms leading to tumorigenesis, tumor progression and metastasis. Furthermore, the JAK/STAT pathway regulates expression of several genes needed for the maintenance of CSC phenotypes and acquisition of drug resistance [86, 87]. JAK/STAT pathway is a potential target for therapeutic purpose to treat several disorders including cancer. Several inhibitors have been synthesized and tested for their activity in vitro and in vivo. Some of the inhibitors are in clinical trials for their application for the treatment of cancer [88].

## OTHER PROMISING THERAPIES

### Phenethyl isothiocyanate (PEITC)

Treating ovarian CSCs with phenethyl isothiocyanate (PEITC) revealed an anti-CSC activity, defined as reduced expression of biomarkers for stemness [89]. Koschorke and colleagues discovered that treating ovarian CSCs with PEITC impaired the spheroid forming efficiency of the cells by decreasing their ALDH-positive compartments. ALDH is a marker for stemness, so this observation implies that the CSCs are being directly targeted and the ALDH^+^ population is decreased resulting in decreased tumor growth and increased responsiveness to chemotherapies [89].

### Graphene Oxide-Silver Nanoparticle Nanocomposites

One study demonstrated the therapeutic potential of graphene oxide-silver nanoparticle nano-composites for ovarian cancer by targeting the ovarian CSCs. Ovarian CSCs were harvested and incubated with the nanocomposite. After 3 weeks of incubation, the number of stem cell colonies were reduced which hinted to the toxicity of the nanocomposite towards the ovarian CSCs [90]. The mechanism for the composite was to generate reactive oxygen species, leaking of lactate dehydrogenase, reduced mitochondrial membrane potential, and increased expression of apoptotic genes, all of which contribute to reduce stem cell viability. Choi and colleagues [90] added salinomycin to the media, which, in tandem with the graphene oxide-silver nanoparticle nanocomposites, and showed an increased rate of apoptosis. This therapy is advantageous in that it requires very low concentrations to be effective, which could result in decreased pricing for therapies along with the conservation of resources [90].

### Verrucarin J and Withaferin A

Recently, Udoh et al [91] and Carter et al [92] showed that a fungal metabolite from myrothecium verrucaria known as “Verrucarin J” (VJ) targets both cancer cells and CSCs in lung and ovarian cancers. Such pronounced effects of VJ were achieved through the inhibition of Wnt/β-catenin and Notch1 signaling pathways. In independent studies, Kakar and his group [93, 94] showed inhibition of tumor growth and metastasis of ovarian cancer by Withaferin A (WFA), a product from *Withania somnifera*, commonly known as Ashwagandha. These investigators also showed targeting of CSCs in ovarian cancer by WFA through the inhibition of transcription of CSC specific genes and those of Notch1 signaling pathways. However, efficacy of these drugs remains to be tested in humans.

Several strides have been performed in order to target ovarian cancer and the CSCs in particular which were recently summarized during the American Association of Cancer Research/Rivkin Center Ovarian Cancer Research Symposium held at the University of Washington in September 2018. Current state of the art of ovarian cancer treatment were highlighted and manifold efforts of researchers towards multiple targeting modalities such as clinical trials employing novel agents, including poly-ADP-ribose polymerase (PARP) inhibitors, other DNA-damaging agents, vascular endothelial growth factor receptor inhibitors, mirvetuximab soravtansine, and immune checkpoint blockade. Innovative novel technologies such as antibody-drug conjugates targeting surface receptors in ovarian cancer either alone or in combination with immune checkpoint blockade exhibited strong translational potential. Potential therapeutic combination partners such as DNA repair inhibiting agents, those targeting cellular checkpoints, and drugs with potential against CSCs were identified. Novel therapeutic strategies based upon endoplasmic reticulum stress response, epithelial to mesenchymal transition, and targeting of surface molecules using novel antibody drug conjugates revealed anti-tumor potential in pre-clinical models and its tremendous clinical potential. Study highlights pronounced the significance of developing effective and combinatorial treatment modalities such as immune checkpoint inhibitors with other immunotherapies, PARP inhibitors, and standard chemotherapeutic regimen, rather than a single drug based treatment modality [41, 95–101]. Besides the primary ovarian tumor, peritoneal tumor microenvironment supporting the ascites derived CSCs and the transition into an aggressive metastatic form of ovarian cancer is implicated rather than the potential primary tumor properties and thus targeting ascites derived CSCs is equally pertinent. Some of the key and novel treatment strategies addressing therapy-induced resistance arising out of putative CSC dormancy and plasticity, highly efficient drug efflux pumps and DNA repair mechanisms have been recently summarized by Ahmed and his group [102].

## CONCLUSIONS

Owing to the complexity of the mechanisms of regulation of ovarian cancer growth and metastasis, there are many opportunities for disrupting these cancer-causing processes. However, there is still significant research to be performed in relation to implementation of these therapies and the best ways to tailor them to individual cases of ovarian cancer. Precision medicine with targeted treatment for ovarian cancer patients based on their individual genetic susceptibility and molecular signatures requires patient stratification and further efforts in terms of clinical studies [101]. The signaling cascades involved in stemness, chemoresistance, and metastasis, such as Wnt, Notch, and Hedgehog, etc. provide many potential points of inhibition of growth of ovarian cancer as aptly reviewed recently by Keyvani and group [103]. In addition, better classification and understanding of CSCs in ovarian cancer, will hopefully lead to better understanding of the origin of ovarian cancer malignancies and even more potential sites of inhibition may be revealed.

## Figures and Tables

**Table 1.** Commonly identified cancer stem cells in ovarian cancer

Marker	Type of protein	References
CD24	Cell surface transmembrane glycoprotein	[31], [104–110]
CD34	Cell surface transmembrane glycoprotein	[31], [111], [112]
CD44	Cell surface transmembrane glycoprotein	[31], [113–116]
CD105	Cell surface transmembrane glycoprotein	[112]
CD117	Tyrosine kinase receptor	[31], [117–122]
CD133	Cell surface transmembrane glycoprotein	[31], [117], [123–127]
EpCam	Cell surface transmembrane glycoprotein	[31], [128], [129]
ROR1	Tyrosine kinase receptor	[37], [130]
ALDH	Cytosolic aldehyde dehydrogenase enzyme	[31], [131–134]
SOX2	Transcription factor	[31–33], [131–134]
OCT4	Transcription factor	[31–33], [80]
NANOG	Transcription factor	[31–33], [138–141]
MYC	Transcription factor	[23], [142]
ABCG1, ABCG2	ATP binding cassette transporter	[143–145]
PTTG1	Cytosolic/nuclear protein	[31]
LGR5	Cell surface membrane protein	[31], [32], [51]
DDX4/VASA	ATP-dependent RNA helicases	[29], [31], [32], [146]
IFITM3/FRAGILIS	Cytosolic protein	[31], [32], [147]
SSEA4	Cytosolic protein	[31], [32]
STELLA	Cytosolic and nuclear protein	[32], [148], [149]

Note: Some of the information are taken from Kenda Suster N, Virant-Klun, I [28]
